# Supplementary material for: Patterns of Intron Gain and Loss in Fungi
Source: PLoS Biol. 2004 Nov 30;2(12):e422. doi: 10.1371/journal.pbio.0020422 (PMC532390; doi:10.1371/journal.pbio.0020422)
Supplement: Table S1 — Also available at http://genes.mit.edu/NielsenEtAl/. (4.3 MB ZIP). [file pbio.0020422.st001.zip › NielsenEtAl/html/1417.html]

AN6598.1.NCU08993.1.MG04982.1.FG06903.1


```
 CLUSTAL W (1.82) Multiple Sequence Alignments - Introns Inserted


Sequence 1: NCU08993.1	2084 aa
Sequence 2: MG04982.1	2099 aa
Sequence 3: FG06903.1	2056 aa
Sequence 4: AN6598.1	2070 aa
Alignment Length: 2141 aa
Number Identitical Residues: 867 aa
Alignment Score (without introns) 46093


MG04982.1 	MSNAWESEREPPSAGEANGHHDDTLAATATPTQPPPRTSAAAANSHHPELDATKLQSLPA
NCU08993.1	MS----STPADATNGERNGITN-TLTSHATTN---------------PELDLAKLQALPA
FG06903.1 	-----MSSTQEPEAPAHNSEAQAPLDTATVN----------------PELDLPKLQSLPA
AN6598.1  	-----MSTAEVARTSSDNNVGNDATPR--------------------AELDITKLHALPS
          	      *    .     *.  : .                       .*** .**::**:

MG04982.1 	DQQELFLLSFTSTLTKHVLALEPDDCTAQQFYLKKEIFQIINLPSPTPSRVIRNNIAKCL
NCU08993.1	EQQELFLLTSVSALSRHVLTLEPDDCTAQQFYIKKEAFKVLNLASPSPTRVIRNALGRCL
FG06903.1 	EQQDLFLLTFVSSLAKHVLSLEADDCTAQQFYLKKEIFQIINLSSPHPSRVIRNNLGKCL
AN6598.1  	EQQDLYLLTFTSDLVQHISTLDKSQVSAEQKFLKRELFKVLTLSSPAITRVIRNNLGRCF
          	:**:*:**: .* * :*: :*: .: :*:* ::*:* *:::.*.**  :***** :.:*:

MG04982.1 	AHVLGTGDRKLLFETINELSALIAATKSKTDGDSKARHAAVVVLGDVFGSAGDSAIGLHQ
NCU08993.1	AHVFDKGDRKLLFETVNELVGIISSGKLKTEGEIRTKHAAVACLGDIYGSAGDSAIGLHQ
FG06903.1 	AHIFEIGDRKLLFETINDLIALVSSGKSKTDAELRTKHAAVVCLGDVFASAGDSAIGLHQ
AN6598.1  	GAIFSKGDRGILFETVTDLLGVLNAG--KNEADLRTKFAAAHCLGDIFTTAGESVFAQSG
          	. ::  *** :****:.:* .:: :   *.:.: :::.**.  ***:: :**:*.:.   

MG04982.1 	MACGNLLKLLKASQNHAGMRSSIFVALSKIVRMIEASLDETLARDIWKQARNHASSDKGS
NCU08993.1	LVCSALLKLLKAASNHAGLRAAVLTAFGKIVTMIQGSMDENVARDIWKQGRNHASGDKGS
FG06903.1 	LACSTLLKLLKASSNHAGLRAACLTALGKIVGMIESSMDEVIARDIWKQGRNHASSDKGH
AN6598.1  	AVVTSLLKLLKPSSNHTGCRGSVFAVLRKVVGGTGVPVDEGTARDIWKQARNAATGDKST
          	 .   ******.:.**:* *.: :..: *:*     .:**  *******.** *:.**. 

MG04982.1 	LVVISACRCVKALVRHTPYFDNSSDFDSLSRTTFKTIDNTSVQARHAAVDCFAEIMVKNY
NCU08993.1	LVVVAACRCLRSLVQYTPYFKNSTDFDKLKSTLFKTFDNPSSNARSAAADCFAQALVGGY
FG06903.1 	LVVASACRTLKVLFKHTPYFLNSGDFDKLESAISKAFDSPSVQVRQAAAECFAEALFQGH
AN6598.1  	FVQVHACRCLEQLLHTTPYFDNANDFESLKTVVWKVIDSPLAPVRHAAAACFARALTKLH
          	:*   *** :. *.: **** *: **:.*. .  *.:*..   .* **. ***. :   :

MG04982.1 	SE--LGAAETAKNKKSRSKSAKRATQAGALQDED-AIPARSESPAPT-KKAQDLALSLTD
NCU08993.1	SESAIGEAPLPLVKKSKSKAIKRQSTMGGLQDDE-EVPSRPESPAPT-QRPQDLTLSLAN
FG06903.1 	SEGGLPEVASPKGKKTK-KALKRQSTIPNIADDELEIPSRSASPAPSGKRSQVLALSLSD
AN6598.1  	AKD-SRIMSTPKPKKAK-RQSKKPAPRPGEDEDE---AEVSESSAPK-KSESRLYFLLPD
          	::.       .  **:: :  *: :      :::   .  . *.**. :  . * : *.:

MG04982.1 	ILKTLSTQYVRSSTSNRARVALGVCHGRVLQRLGEKTVEINFIKLIEALSNDILGHSNIS
NCU08993.1	ILKTLSTQYVRISTTNKARAAIAICIGNVLQKLGEKTVEVNYLCILEILTADFLGHNNIL
FG06903.1 	ILKNLASQYTKSTASNRTRAAIGVCYGKLFRRLGEKVVETNYLKIVESLAIDVLGYPAIA
AN6598.1  	LLKQLSQQYLRSTTSNRARAGIAVCYKYVLRILGEKLVEERYDQIANHLLFDLLNHPTIT
          	:** *: ** : :::*::*..:.:*   ::: **** ** .:  : : *  *.*.:  * 

MG04982.1 	NSRYKLLASRRIVDTIIQDMVGKNILGESGQASAAKAIINDILKNYPQSLKELSEPSKNT
NCU08993.1	NNRYRLLISRRMVDTIIQDILGKRILGESGQITAAKAIVNDILKNYPQALKEKPEPSKHT
FG06903.1 	NNRYKLLVSRKIVDVVVQDVIGRKILGESGQISAAKSLINNIFKNYPQALAERPEPSKHT
AN6598.1  	NNRFRLLMTRKVVGSILEETVGRDSLRENSRLTAAKWLINDVLKDYPQVVQERREPSKYT
          	*.*::** :*::*. :::: :*:  * *..: :*** ::*:::*:*** : *  **** *

MG04982.1 	LIAALGALASLIQSLGSAANNFAEACRDALLQVLQHPSYSVQVFASSCMKTFVLACPQQL
NCU08993.1	LVVSLSALSSLISSLGTAVTDFSEGCRDGLLQVLQHPSYTVQVYAAACMKNFVLACPQQL
FG06903.1 	LIATLSAVSSLIQSLGSAANTFAESCRDGLLQVLQHPSYTVQVFASHCMKTLVLACPQQL
AN6598.1  	LINALNALSSLVSSLGSAFGSLAESCRDALLQVLSHPSYTVQIHAAHCLRIFVLACPVQL
          	*: :*.*::**:.***:*   ::*.***.*****.****:**:.*: *:: :***** **

MG04982.1 	LPCLSVCMNSLGRELGLLGTGRNSPRRCVGFAHGLAAALSASPLRPLHGSVEINSRVLNM
NCU08993.1	LPCLSVCMNSLSRELSLLGTGRNSPRRCIGFAHGLAAGLSASPQRPLYGSVEINSRVLTM
FG06903.1 	LPCLSVCMNSLSRELSQLGSGRNSPRRCIGFAHGLAATLSASPARPLHGSVEINSRVLTI
AN6598.1  	LSCVTICLNSLNREVGQLSTPRQSPRRCVGYANGLSAMLSTSRLQPLYGSVEIYSRVFTQ
          	*.*:::*:***.**:. *.: *:*****:*:*:**:* **:*  :**:***** ***:. 

MG04982.1 	ATNLLKSSGTMELRVASTQIQVAWTLIGGLMSLGPNFVKIHLSQLLLLWKNALPKPLAKD
NCU08993.1	ATNLLKSSGTSELRVASTQIQVAWTLIGGLMSLGPNFVKIHLSQLLLLWKNALPKVLAKD
FG06903.1 	ATNMLKSSSQAQLRVASTQIQVAWILIGGLMSLGPNFVKIHLSQLLLLWKNALPKPLAKD
AN6598.1  	ATDLLKTSSSAELRAASTQIQVAWILIGGLMPLGPSFVKIHLSQLMLLWKNALPKHLSQE
          	**::**:*.  :**.********* ******.***.*********:********* *:::

MG04982.1 	-NSSSRNYLEVSFLTHVRECALGSILAFLEFNSRLLTVDVSKRIATMLQHTTAYLKTLPA
NCU08993.1	SMAVHRNLLNASFLTHVRECALGSILAFLQFNSRLLTVDVCKRIATMLQSTTAFLKTLPA
FG06903.1 	-NTSARSFLEVSFLTHVRECAMGSILAFLEFNSRLLTVDVSKRIATMLQNTTAFLRTLPS
AN6598.1  	-NSAKKGYLEMSFLAHVRECALGALLAFMEFNGKMITADGARRIATMLQNTVEFIDEIPP
          	  :  :. *: ***:******:*::***::**.:::*.* .:******* *. ::  :*.

MG04982.1 	KRTTDDISQRLAPALQLYDLDMMVSRRVLQCYTKLINLSPPGGTEALLQSNLLTLAISLF
NCU08993.1	KKTSDDISERLTPALQLQDLEVMVRRRVLQCYTKLVNVSPAGATEALLQSNLLTLAISLF
FG06903.1 	KKNSDDVNQRLAPSLQLSDLEMMVQRRVLQCYVKLVNLSPAGGSESLLQSNLLTLAISLF
AN6598.1  	QKSVVDISQRLHPSLQLHDIETMVRRRVLQCFAKLLHAHPLSHADVISQSSLLSLAISSF
          	::.  *:.:** *:*** *:: ** ******:.**::  * . :: : **.**:**** *

MG04982.1 	ADPDNYTPSSLSATIANAAGAFDTIWDVGDNAGFGVTGLVRGFSVKPLPGQHENSTEPSP
NCU08993.1	ADPDNYTPSSLSTSIANAAGTFDSIWEVGDNSGFGVTSLVRGFATRSLPGQQTNGSD-ST
FG06903.1 	ADPENYAPNSLSASIANAAATFESIWDVGDNSGFGITGLVAGPRVRKLPGQQENSDDPRD
AN6598.1  	ADPDATQSSPLESSIAASTAQFENLWELSDNYGFGVTGLARDYVRVTLSGKHEGESGPAW
          	***:   ...*.::** ::. *:.:*::.** ***:*.*. .     *.*:: .   .  

MG04982.1 	IDESGPEAAMEKL~LMSPVCGSLEHDASVLYIGQYQPDSGLESLPDPPATEVVNVAIQLF
NCU08993.1	QQEYEPEECIERL~LLSPVCGTLENDACLLYIG----NSDNPGLPDPAATEVVNMAIQLF
FG06903.1 	EFDS-PDDTINNL~LRSPICGSLEHDASVLYIG---STDHSSTTPDPPATEVINAAIQLF
AN6598.1  	SAVESTDQVVDDA0LIFPICEASEHDPILLYSAR----QGDRLSADPHPTGVVNAAIELF
          	     .:  ::   *  *:* : *:*. :** .     .     .** .* *:* **:**

MG04982.1 	AFVLPLTPPKVQESILEQITTFLSAGSLQRETGRKAAINVNIAIAILYTLKVAVKETQAP
NCU08993.1	AFVFPLTPSKVQESALEQITTFIRAGSLQRETGRKAAVDVNVVTALLSTLRVACKETKSS
FG06903.1 	AFAFPLTPGKVQESILEQIRTFASAGTLIRDPGRKAAMNVNLAVAILSTMRVAVKETKSP
AN6598.1  	SVALALHAPKVQESSVEQIATFLSSTGLQRNPGRKAAMVVNISVALLHALKVAMKETSSA
          	:..:.* . ***** :*** **  :  * *:.*****: **:  *:* :::** ***.:.

MG04982.1 	RGDVTNMAVEKLLQEMIR~DFVLSPDQYVRSIGYAAVARLCSTCGNAFTNHEIKYLVDTI
NCU08993.1	PGDITNMAVEKLLQELLR~DFVLDPDQYVRSIAYEAIARLCSACGNAFTNQEIKFLVDTI
FG06903.1 	SGDVSNQAVEALLQDIVR~DFVLDSDEHVRSLGYAATARLCNVYGNAFTNNEIKYLVDTI
AN6598.1  	ARKLS-PTTEKVMQELIQ0KFVLDPDPIVRTVGVEALGRLCESSGNTFTNTQINWVVDTI
          	  .::  :.* ::*:::: .***..*  **::.  * .***.  **:*** :*:::****

MG04982.1 	VINREPSARAGCAMALGCIQTKVGGMAAGYHLKTILGIIMSLCNDPHPTVHFWALEAFSR
NCU08993.1	VVNREPSARAGCAMALGCIQTKIGSMAAGYHLKTILNILMSLCNDPHPTVHYWALEAIAR
FG06903.1 	VGNREPSARAGCAMALGSIQTQVGGMAAGYHLKTILGILMSLCNDPHPVVHFWALEALSL
AN6598.1  	VANREPNARAGCAAALGCIHSQIGGMAAGLHLKTIVGVLMSLGNDPHPVVHFWALGGLEK
          	* ****.****** ***.*::::*.**** *****:.::*** *****.**:*** .:  

MG04982.1 	MADAAGLGFSAYTSSTLGMLAQLYVSDNHNAETGSAVSMSLEVELCTPGAIARGIDSIIN
NCU08993.1	VSDAAGLGFSSFVSSTLGMLAKLYVSDTHNPELASPITMNLEADLSSSAAIARCVDALIN
FG06903.1 	ASDASGLGFAGYVPSTLGMLVQLYVSETHHAEISSAITMNIEMDLSTTSAIARCVDSLIN
AN6598.1  	VANSAGLTFSPFVSSTLGMLAQLYNADSHNEEAESLATSNIEMSFLTPVVISRCIDSLIN
          	 ::::** *: :..******.:** ::.*: *  *  : .:* .: :. .*:* :*::**

MG04982.1 	VLGPDLQDSTKSRELIFDLVGQFIGEEDSQVQLVALSCMEHLSMFAPGFVDFKDYVRLLQ
NCU08993.1	VLGPDLQDSTKSRELILTLVGYFQQEEDLEIQRASLACMEHLTLYAPGYMDFIDYVRTLQ
FG06903.1 	VLGPDLQDALKSRELIFTLVNRFREEDDLLVQRAALGCLEHLSLYAPGHMHFADYVKILQ
AN6598.1  	VLGPDLQDIAKTRNLILTLLRQFQLEENPALVTESSRCLDHLSLYAPEYVDFSGYVKRLQ
          	********  *:*:**: *:  *  *::  :   :  *::**:::** .:.* .**: **

MG04982.1 	KHINSKHPSLRDAAVDGLYSLMKRDSKDVIQAAEKGFEDELWLVLDTCPSHDGIRNLVRN
NCU08993.1	RYIKSDHAALRDVAVDSLYNLMKRNSYDVIKAAEQGFEDHLWLVLDASPSHDGMRNLIRN
FG06903.1 	GYLMSESTTLRDVSVDGIHNMMKRNPQDVLREAETGFEEQLWLVLDTAPSHDGIRNIIRN
AN6598.1  	NELSAQDPLMRDVAIQGLTNLMKRDAPAVIKAATPALEEEIWLAFDDNPDNPILKGMIRD
          	  : :. . :**.:::.: .:***:.  *:: *  .:*:.:**.:*  *.:  ::.::*:

MG04982.1 	WLQQTCLSDTTGWLQRLQGVLKMTRTKVVETAKVSTGRSNAIIDLQDEEVAGFA-AAGGV
NCU08993.1	WLRQTCLSDTAAWLARFQHVLKMTRPKASAQATTNTGRSGGGIDLQDEEVAGFA-AAGGG
FG06903.1 	WMHSTCLAETNAWLQRFQAVLKMTRARPKTEEAQSGSRGGGLPDLQDEEVAGFA-AAAGA
AN6598.1  	WLQQTALTETGLWIQRCHDTLTKTRLKVEDLPPISALKP-TATDLPDDEVAGFASAIAGA
          	*::.*.*::*  *: * : .*. ** :       .  :     ** *:******:* .* 

MG04982.1 	AKDDKESAGGSDVEALRWQVMTFVMSCLNDIFTLISKEVATHGESAAQTALQNKVADVVR
NCU08993.1	AKDDKDTPSGSDVEPLKWQVTTFAMDCLNDIFILVTKDVATHGESAAQAALQNKVADVVR
FG06903.1 	AKDEKETKATSEVEPLRWQVTTFALSCIDEMFAIIAKDVTANGESKAQNDLQSRIADVVR
AN6598.1  	GQEDGAGDAVAGQELLKWQTRNFVMSCLSELLELVQEAILPDQTIPAELALQQKVGDIVR
          	.:::    . :  * *:**. .*.:.*:.::: :: : : ..    *:  **.::.*:**

MG04982.1 	MAFSASTAGVLEQRIWGLKIIGSVLK~MFGKTPDPDFEEAMLLEQYQAQISSALTPAFAA
NCU08993.1	MAFSASTSGVLDQRIWGLKIIGAVLK~MFGKIPDPDFEEAMLLEQYQAQISSALTPAFAA
FG06903.1 	MAFSASTSSVLELRIWGLKIIGDVLK~MFGKTPDPDFEEAMLLEQYQAQISSALTPAFAA
AN6598.1  	MAFSASTANVIELRVWGLKILDQVLK0MFGKTPDPDFAEASLLEQYQAQIGSALTPAFAA
          	*******:.*:: *:*****:. *** **** ***** ** *********.*********

MG04982.1 	DSSPELASEAVNVCAAFIATGIVTDVDRMGRILKTLVAALENFATEKENVGIGELKGLSS
NCU08993.1	DSSPELASEAVDVCASFIATGIVTDVDRMGRILKTLVSSLENFAKDEDNAGIGDLKGLSS
FG06903.1 	DSSPELASEAVNVCASFISIGIVTDVDRMGRILKTLVGALENFSKDSENAAIGDLKSLSS
AN6598.1  	DSSPELASEAINVSATFIATGIVTNVERMGRILKLLVLGLENFSANPDTTEIGDLKGLNS
          	**********::*.*:**: ****:*:******* ** .****: : :.. **:**.*.*

MG04982.1 	NAQVMVKMSVFAAWAELQVASSEQKYLFEVLKSHIGTLTPLWLESLREFARLRFEPDISM
NCU08993.1	NAQVMVKMSVFAAWAELQVASSEQKYLLDVLKPHIGTLTPLWLESLREFARLRFEPDISM
FG06903.1 	NAQVMVKISVFSAWAELQVASSEQHYLLDVLRPHIGTLTPLWLESLREFARLRFEPDISM
AN6598.1  	NAKVMVKMALYAAWARLQIASIEHEYLNEVVQPYLLKLTPLWLSSLQEYARLRFEPDISG
          	**:****:::::***.**:** *:.** :*::.:: .******.**:*:********** 

MG04982.1 	TLGPPSLSGSLDTIYAALNRETLLK~FYQESWLKLVDAIASLIEQDSEFVFDALDG----
NCU08993.1	TLGPPSLSGSLDTVYAALNRETLLK~FYQESWLKLVDAIASLIEQDSEFVFDALDG----
FG06903.1 	TLGPPSLSGSLDSIYAALNRETLLR~FYQDAWLKLVDAIASLIEQDSEFVFDALDG----
AN6598.1  	SLGTGPESGDLDEVYAALNRETLLK0FYQETWLSLVDAIAGLVERDIDFVFDALDGKAQI
          	:**. . **.** :**********: ***::**.******.*:*:* :********.:. 

MG04982.1 	----KETDGPASTSKGKAKSADINYRDEPVAFFFVLFGIAFEALAAKPGQPDSLATQAQT
NCU08993.1	----KELSGPTTNSS--SKGADINYRDEPVAFFFVLFGIAFEALATRPGQSESLATREQT
FG06903.1 	----KESDGPAASGK--SKSSDINYRDEPVAFFFVLFGLAFEALATRSGQNDSLAAHDQT
AN6598.1  	KEPEKSEEDQLSNGEPKGKGDDINYRDEPVAFFFVLFGLAFEALVDQS------TSASQR
          	....*. ..  :... ..*. *****************:*****. :.      ::  * 

MG04982.1 	LEILRALRKILHPSVSGHAIYREAIFTEAMDLLDRLVLTEGLDVQAVIVQIARALCVAHP
NCU08993.1	LEILRALKKILHPSVSGHAIYRDAIFSETMDLLDRLVLTEGLDVQGVIVEIARALCVAHP
FG06903.1 	LAILQALKKILHPSVSGHAIYRPDVFSETMDLLDRLVLTESLDIQGVIVEIARSLCIAHP
AN6598.1  	MEILQALKRILRPIISGNAIYQDAIFSETMDSLDRLALTEGTAVQNVIVEIARNLALDHP
          	: **:**::**:* :**:***:  :*:*:** ****.***.  :* ***:*** *.: **

MG04982.1 	AARKRADQADSGELSEDIEQLFELTRIIVLVLSGLLPNLTTDAASGGQQPSAVRHQMTEE
NCU08993.1	AARKQADDPENSELSDDIEQLFELTRIIVLVLSGLLPNLS-----ETQQP--VRHQMTEE
FG06903.1 	SARRHAN--EEGDLSDDIDQLFELTRIIVLVLTGLLPNLS-----EGNQP--ARHQMNEE
AN6598.1  	SAKGGED--RSDHLSDDIEQLFELTRSIILVLAGLLPNLR------ESTTIARFNVGSDD
          	:*:   :   ...**:**:******* *:***:******        . . :  :  .::

MG04982.1 	AITLVRTSLNALVDAAEVFPAIIRTDLHACIIHIFATILATPACQQIIVPQSLPTLKRFI
NCU08993.1	AILLVKTSLNALVDAAEVFPSIIKTDLHACILHIFATILATPSCQEVIVPQNLSTLKRFI
FG06903.1 	AILLIQSSLNALVDAAEVFPAIIKTDLHACIVHIFATILSTPGCQELIAPQSLPTLKRFV
AN6598.1  	ALSLIRLSLSSLVNVASIFPSIIRNDLNACILHIFSTILATGLCQQEVVPQALPIFKHFI
          	*: *:: **.:**:.*.:**:**:.**:***:***:***:*  **: :.** *. :*:*:

MG04982.1 	GISSHSQQPGAEGGDGSGSQTTAQLQGCLRRFLSIYLHAQKREAPTSLSCVKNCLLAITI
NCU08993.1	NGMTGSRRDGGDDDEGN-SPTDVQLLGCLRRFLSIYLNAQKREAPTSLTCVKNCLLAITI
FG06903.1 	GSMSKSRRS---HSNGEPSSTDIQLQGCLRRFLSIYLNAQKRESPQSLTCVKNCLMATTI
AN6598.1  	QSISGQENASLENQENQ-NVISHQLRGCLVRFLTTLTIAQRRESESSLPCAKNTLLTITI
          	   : ... . .  :.. .    ** *** ***:    **:**:  **.*.** *:: **

MG04982.1 	LFTGGKNYMSANDPLVAKFLDEVVDCLSDRM0TAKIAANCIRSLLVQAN----PTAADQS
NCU08993.1	LFTGGKNHLPASEPLVAKFLDELVDCLTDRM0TAKIAANCIRSLLLNTNANNQPTSADHS
FG06903.1 	LFTGGQNHLPASEALVARYLDELMDCLTDRM0TAKIAANCVRSILLQPS----STGADLS
AN6598.1  	LLTTGGHLIPPHDPILPQILNELLDCLQDVG~LASVAAGCIRSILLSNS---TKSNTDEV
          	*:* * : :.. :.::.: *:*::*** *    *.:**.*:**:*:. .     : :*  

MG04982.1 	IARYLLPRLVVFVTNTEPE-------DPERARALVSHALTSFVAAVPR----DHGDAVAV
NCU08993.1	ISRHLLPRLIAFVTNTSPE-------DPEQARSLIAQTLVQFTAVVARGSSQDGGQGASI
FG06903.1 	IARYLFPRLVAFVTDTDPE-------DPENARALVAHTLCQYAGTVDK-------GRRLA
AN6598.1  	IARYLIPRLIAFLIGTPTDPGGEASNDPENSRTSIARTLVSCVTVPNAAA--FPGGETHT
          	*:*:*:***:.*: .* .:....::.***.:*: ::::* . . .   .:    .     

MG04982.1 	AMSLVMPALLARAAAEEEDEGEEGTVYKETSACLLQLASVDQKFFRGVVAGMSEGQKAFM
NCU08993.1	AMALVVPTLLSRAAT----EGAKDDAYRETSARLLELASADQTAFRAVVGAMSEGQRAFM
FG06903.1 	AITVVVPTLMARATA----EGEE--VYQETSARLLELAATDQEVFRAVVGGMSDGQKGFL
AN6598.1  	AMSLVMSCLLARAKR----EGAP--VYTETAANLLELAKADQLAFRTLVGTMSPALKGLL
          	*:::*:. *::**      **    .* **:* **:** .**  ** :*. ** . :.::

MG04982.1 	EEVIRSGRRTAQEKDSGAASG-------------QPSIALKMDFGG
NCU08993.1	EEVIKSGRSGGMGGGGGGAHVGAGGRRSEEDEEQAPTIALKMNFGG
FG06903.1 	EEVIRFGRQNTSQVSKAAAGDSG-----------QPTIALKMNFGG
AN6598.1  	EEILRNANVGAGGGAVGRGEQETQ---NGGGQQSMPSIALRFDF--
          	**::: ..        . .        .  .... *:***:::*
```
